# Special series on “effects of board games on health education and promotion” board games as a promising tool for health promotion: a review of recent literature

**DOI:** 10.1186/s13030-019-0146-3

**Published:** 2019-02-19

**Authors:** Mutsuhiro Nakao

**Affiliations:** Department of Psychosomatic Medicine, School of Medicine, International University of Health and Welfare, 4-3, Kozunomori, Narita-shi, Chiba-ken 286-8686 Japan

**Keywords:** Board game, Chess, Dementia, Go, Lifestyle modification, Shogi

## Abstract

Board games are played by moving game pieces in particular ways on special boards marked with patterns. To clarify the possible roles of board game use in psychosomatic medicine, the present review evaluated studies that investigated the effects of this activity on health education and treatment. A literature search conducted between January 2012 and August 2018 identified 83 relevant articles; 56 (67%) targeted education or training for health-related problems, six (7%) examined basic brain mechanisms, five (6%) evaluated preventative measures for dementia or contributions to healthy aging, and three (4%) assessed social communication or public health policies. The results of several randomized controlled trials indicated that the playing of traditional board games (e.g., chess, Go, and Shogi) helps to improve cognitive impairment and depression, and that the playing of newly developed board games is beneficial for behavioral modifications, such as the promotion of healthy eating, smoking cessation, and safe sex. Although the number of studies that have evaluated board game use in terms of mental health remains limited, many studies have provided interesting findings regarding brain function, cognitive effects, and the modification of health-related lifestyle factors.

## Introduction

Board games are played by moving game pieces in particular ways on special boards marked with patterns [1]. For example, one game originated in northern India in the sixth century AD and spread to Eastern as well as Western countries. In the West, it spread to Persia and then to Spain via the Moorish conquest, and then throughout Europe, where it ultimately became “chess.” In the East, this game became “Xiangqi” in China, “Shogi” in Japan, and a variety of similar games in other countries. Other popular board games that use two patterns for the game pieces include “Go” and “Othello,” also known as “Reversi.”

In the field of psychosomatic medicine, board game playing is sometimes regarded as a leisure activity, and engagement in this type of activity has been shown to protect against dementia and cognitive decline in elderly individuals [2]. For example, a 20-year prospective population-based study conducted in southwestern France investigated the relationship between the playing of board games and the risk of subsequent dementia [3]. Of the 3675 participants without dementia in that study, 1176 (32%) reported regular board game playing and 840 (23%) developed dementia during the follow-up period. The risk of dementia was 15% lower in board game players than in non-players, and board game players exhibited lesser declines in Mini-Mental State Examination (MMSE) scores and less incident depression than did non-players. Although the mechanisms underlying the reduced risk of dementia in board game players have yet to be elucidated fully, these games require players to be proactive and to anticipate, thinking several steps ahead, during play. These processes may enhance logical thinking and prevent declines in cognitive function. Individuals may also engage in non-verbal communication while playing board games, and players are more likely to have the opportunity to gather and participate in a fun activity with others. These factors could enhance individuals’ social networks, which also protects against cognitive decline. Furthermore, in terms of leisure activities, board game playing may also be a form of stress management [4], as the fight-or-flight response is regulated safely within the sophisticated structures of match-type games. Board game playing could also be a form of art therapy, similar to miniature garden therapy [5], facilitating infinite internal manifestations within a narrow space.

In terms of education, the playing of board games may help children learn to follow rules and stay seated for a certain amount of time, and it may increase children’s concentration levels [6]. For students and trainees, board game use can enhance health education by stimulating players’ interests and motivation. A search of the Cochrane Database of Systematic Reviews [7] identified a total of 2079 unique citations related to educational games, such as board games and games based on television shows. Of these citations, 84 were potentially eligible for review based on methodological quality, number of participants, interventions, and outcomes of interest, and two randomized controlled trials (RCTs) were chosen. The first study [8] was based on the television game show “Family Feud” and focused on infection control; the group that was randomized to play the game had significantly higher scores on a knowledge test. The second study [9] compared game-based learning (using “Snakes and Ladders”) with traditional case-based learning of stroke prevention and management information. Although the two study groups did not have significantly different knowledge test scores immediately or 3 months after the intervention, the reported level of enjoyment was higher in the game-based learning group. The findings of an original review of articles published through January 2012 [7] neither confirmed nor refuted the utility of game playing as a teaching strategy for health professionals. Thus, the present study aimed to clarify the possible roles of board game use in psychosomatic medicine through a literature search for articles published after 2012 that focused on the effects of board game playing on health-related issues.

## Mind/body changes due to board game use

Using “board game” as a PubMed search term, 83 studies published between January 2012 and August 2018 were identified; 56 (67%) articles targeted education or training for health-related problems, six (7%) examined basic brain mechanisms, five (6%) evaluated preventative measures for dementia or contributions to healthy aging, and three (4%) assessed social communication or public health policies. The major studies that investigated the effects of traditional board game use are shown in Table 1 [10–33]; some of the articles listed in the table were identified in the reference sections of the original 56 articles or other databases.

**Table 1:** Examples of recent studies using traditional board games

Authors (years)	Countries	Study design	Subjects or materials	Outcomes or variables	Impact
Chess:					
Fuentes JP et al. (2018) [10]	Spain	Experimental, single case	Expert chess player, male, 33 years old	EEG changes, decreased heart rate variability	Increased cortical arousal by critical flicker fusion threshold, decreased heart rate variability during chess play
Barzegar K & Barzegar S (2017) [11]	Iran	Clinical case	Middle-aged man with panic attack after post-traumatic stress	Clinical course, including subjective physical symptoms	No symptom of nausea, vomiting, or panic attack after cell-phone chess play
Schaigorodsky AL et al. (2016) [12]	Argentina	Database	1.4 million chess games played by humans	Long-range correlations, inter-event time distributions	Cattuto’s model well described long-range memory used in opening chess lines
Chassy P & Gobet F (2015) [13]	UK	Database	667,599 chess games played by experts from 11 civilizations	Conflict avoidance, risk-taking behaviors during open aggression	Buddhist experts used riskiest strategy nearly 35% more vs. Jewish experts
Sheridan H & Reingold EM (2014) [14]	Canada	Experimental	41 chess players (17 experts, 24 novices)	Eye movements in 8 chess problems	Only experts distinguished relevant and irrelevant information during early trial
Moxley JH & Charness N (2013) [15]	USA	Meta-analysis	4 studies of age and skill effects in chess	Age, chess skill, move selection, chess recall	Best-move, recall tasks associated negatively with aging, positively with skill
Leone MJ et al. (2012) [16]	Argentina	Experimental	25 chess games played by 9 subjects	Heart rate variation	Heart rate signals relevant cognitive episodes, e.g., objective choice correctness events
Go:					
Barradas-Bautista D et al. (2018) [17]	Mexico	Computer simulation	Ising Hamiltonian model of black, white Go stones fighting	Two-player scenarios, cancer vs. immune system	Go, Ising model provided elements for characterization of cancer invasion, reduction, metastasis
Bae J et al. (2017) [18]	Republic of Korea	Questionnaire survey	63 subjects predicting outcome of AlpaGo vs. Sedol Lee match	Network density, game predictions	Game predictions more accurate in low-density vs. high-density group
Silver D et al. (2016) [19]	UK	AI Go program	Search algorithm of Monte Carlo simulation and networks	Go win rate	AlphaGo had 99.8% win rate against other Go programs, defeated human Go champion
Lin Q et al. (2015) [20]	China	RCT	147 patients with Alzheimer’s disease	Cognitive impairment, depression, anxiety, serum BDNF level	Go ameliorated Alzheimer’s disease symptoms, with BDNF up-regulation
Kim SH et al. (2014) [21]	Republic of Korea	Case-control study	17 children with ADHD, 17 age-, sex-matched controls	Cognitive function, brain EEG changes during Go play–based education	Right theta/beta change in prefrontal cortex during study period greater in ADHD group
Jung WH et al. (2013) [22]	Republic of Korea	Experimental	17 Go experts	Structural, functional MRI during working memory tasks	Experts had increased gray-matter volume, functional connectivity around amygdala
Lee MK et al. (2012) [23]	Republic of Korea	Clinical case	11 patients with reflex epilepsy, including 6 male Go players	MRI, EEG with clinical course	Individualized strategies like game avoidance most effectively prevented seizures
Shogi:					
Tanaka K (2018) [24]	Japan	Review	Summary of data from [26, 27, 30]	fMRI changes in game situations	Cingulate cortex essential for intuitive, strategic decision making for any given Shogi board position
Nakao M et al. (2017) [25]	Japan	Protocol, RCT	65 men aged ≥65 years	Cognitive-behavioral attitudes, depression, anxiety, well-being	Depression, anxiety levels lower during 6-week Shogi stress management program
Wan X et al. (2016) [26]	China	Experimental	17 professional, 17 amateur Shogi players, 19 novices	fMRI signals during problem-solving tasks	In professional group, rostral frontal cortex activated only in post-decision period
Wan X et al. (2015) [27]	China	Experimental	17 amateur Shogi players	fMRI signals during quick offense-vs.-defense strategy decisions	Rostral anterior, posterior cingulate cortices encoded defense, attack strategy values
Nakanishi H & Yamaguchi Y (2014) [28]	Japan	Experimental	12 professional, 12 amateur Shogi players, 12 novices	EEG responses in quick understanding of Shogi game patterns	Frontal area responded only to meaningful game positions, in contrast to temporal area
Aoyagi M & Ogawa T (2013) [29]	Japan	Clinical case	Man with Alzheimer’s disease aged 75–79 years	Frequent chewing for aspiration pneumonia prevention	Shogi play encouragement useful for education about frequent, smooth chewing during eating
Wan X et al. (2012) [30]	Japan	Experimental	20 men aged 20–22 years with little Shogi knowledge	fMRI changes during Shogi training period	Activation in caudate nucleus head developed over training course
Others:					
Duan X et al. (2014) [31]	China	Experimental	20 expert Chinese-chess players, 20 novices	Functional connectivity networks assessed by fMRI	Increased connectivity between basal ganglia, thalamus, hippocampus and parietal, temporal areas in experts
Panphunpho S et al. (2013) [32]	Thailand	RCT	20 elderly Ska players, 20 elderly controls	Cognitive function (memory, attention, executive function)	16-week Ska group joiners had significantly better cognitive function scores
van den Dries S & Wiering MA (2012) [33]	The Netherlands	Computer algorithms of learning to play Othello	Combination of three structured neural network techniques	Evaluation functions (simple linear networks, multilayered perceptions)	Method outperforms linear networks, fully connected neural networks or evaluation functions evolved with algorithms

Experimental studies investigating brain magnetic resonance imaging (MRI) or electroencephalographic (EEG) signals in professional board game players [10, 22, 26–28, 31] demonstrated that the basal ganglia play an important role in the ability to rapidly determine, or intuit, the best subsequent move in a game situation [24]. Additionally, variations in heart rate and eye movements were examined as physiological parameters during chess play [10, 14, 16]. In case studies and case-control studies, board games were shown to effectively improve symptoms in individuals who experience panic attacks [11], as well as those with attention-deficit/hyperactivity disorder (ADHD) [21] and Alzheimer’s disease (AD) [29]. On the other hand, one study revealed possible hazardous effects associated with the playing of “Go” in individuals with seizure disorders [23]. The amounts of real and virtual playing of board games have increased recently and, as a result, the number of published studies assessing the effects of board game use has also increased [12, 13, 17, 19]. The increase in game play is likely due to the prevalence of computer systems in the current age of information and communication technology (ICT) and artificial intelligence (AI).

## Recent RCTs evaluating board game use

According to a recent meta-analysis of four studies that investigated chess play [14], age and skill have differential effects on two tasks during game play: selecting the best move for chess positions and recalling chess game positions. The authors found that age was associated negatively, whereas skill was associated positively, with performance in both tasks. Another RCT showed that an intervention using Go improved depression and increased serum levels of brain-derived neurotrophic factor (BDNF) in patients with AD [20]. Similarly, players’ depression and anxiety levels were shown to decrease significantly during a 6-week stress management intervention that utilized Shogi games [25]. Although these data have been presented only at a scientific conference, they will soon be published in this special series. The authors also reported that several patterns of negative cognitive distortion (e.g., lower levels of activity) significantly improved following completion of the Shogi program compared with those in a wait-list control group. An RCT showed that the playing of “Ska,” a traditional board game in Thailand [32], enhanced cognitive function in terms of memory and attention in elderly subjects.

Of the 83 articles identified in the present PubMed literature search, 12 articles [34–45] report on RCTs that assessed non-traditional board games (Table 2). A variety of board games has been developed to aid in the health education of patients, children, and medical trainees; most of these games are focused on behavioral modifications, such as the promotion of healthy eating [34, 38], smoking cessation [43], and safe sex [45]. For example, in a Swiss study [43], 240 current smokers were assigned randomly to a group participating in smoking cessation program employing an educational board game (“Pick-Klop”) and a wait-list control group. Compared with those in the wait-list group, individuals in the board game group were less likely to remain smokers at the end of the program and at the 3-month follow-up assessment. The authors suggested that use of the board game would be an interesting alternative for the education of smokers in the precontemplation stage.

**Table 2:** Examples of recent RCTs using board games

Authors (years)	Countries	Subjects	Board games	Control setting	Outcomes or variables	Impact
Nederkoorn C et al. (2018) [34]	The Netherlands	66 children aged 3–10 years	Age-appropriate memory-related board game	Play with large bowl filled with colorless, odorless jelly (Jelly group)	Acceptance of a food with a specific texture	Jelly group ate significantly more jelly dessert
Fancourt D et al. (2016) [35]	UK	352 subjects aged > 16 years without surgical training	Board game requiring removal of 3 organs from Cavity Sam (experimental tool)	Operating theater sound, classical music, or rock as background music	Surgical speed, accuracy, and perceived distraction	Rock music impaired men’s performance of complex surgical procedures in board game
Karbownik MS et al. (2016) [36]	Poland	124 medical students	AntimicroGAME to learn bacteriology, antimicrobial drug actions	Lecture-based seminar	Short-term knowledge retention about pharmacology of antimicrobial drugs	Long-term knowledge retention greater in board game participants vs. controls
Sharps M & Robinson E (2016) [37]	UK	143 children aged 6–11 years	Board game with descriptive social norm–based or health message	Board game with animal images	Children’s fruit and vegetable intake	Health and social norm–based messages increased fruit and vegetable intake vs. controls
Viggiano A et al. (2015) [38]	Italy	3110 subjects aged 9–19 years	Kaledo board game to promote nutrition education, improve dietary behavior	No board game during study period	Adolescent food habits and body mass index	Treatment group showed improved nutrition knowledge, healthy diet, food habits, physical activity
Fernandes SC et al. (2014) [39]	Sweden	125 children aged 8–12 years	Educational board game, video, or booklet with surgery and hospitalization information	Entertaining tools with same formats (comparison group), no tool (control group)	Children’s preoperative worries and parental anxiety	Educational group less worried about surgery, hospital procedures vs. other two groups
Laski EV & Siegler RS (2014) [40]	USA	42 kindergartners, mean age 5.8 years	Numerical board game, counting on from current number on board	Same game, standard count-from-1 procedure	Children’s knowledge of numbers in the 0–100 range	Number line estimates, numeral identification, count-on skill improved more in count-on group
Charlier N & De Fraine B (2013) [41]	Belgium	120 students	Board game to obtain first-aid knowledge	Traditional lecture	Students’ Knowledge of first aids	Game condition was preferred, but lecture more effectively increased knowledge
Swiderska N et al. (2013) [42]	UK	67 medical students	Educational board game in neonatology	Normally provided teaching	Students’ test scores in neonatology	Neonatology test scores higher in game vs. control group (*p* = 0.09)
Khazaal Y et al. (2013) [43]	Switzerland	240 current smokers aged 18–65 years	Pick-Klop game, cards with smoking-related questions, response options	Psychoeducation to stop smoking, wait-list control	Smoking-related attitudes and behaviors	Game group less likely to remain smokers vs. wait-list group
Cho KH et al. (2012) [44]	Republic of Korea	24 stroke patients	Virtual reality training with balance-board game system	Standard rehabilitation program only	Statics balance of chronic stroke patients	Significant improvement in dynamic balance in chronic stroke patients with virtual-reality balance training
Wanyama JN et al. (2012) [45]	Uganda	180 HIV-positive participants	Educational board game to impart health knowledge	Standardized health talk	Uptake of knowledge to HIV and sexually transmitted infections	Educational game improved uptake of HIV, sexually transmitted infection knowledge

## Clinical applications of board games

Based on the results of studies investigating traditional and non-traditional board games, it was hypothesized that board game use would prevent cognitive impairment in elderly individuals and illness-prone behaviors in children and adults. Board game playing also seems to be an effective, fun means of delivering medical and safety education to students and trainees. Currently, many people spend large portions of their time playing games online and offline on television monitors, personal computers, tablets, and/or smart-phones. For example, more than half of Japanese elementary and junior-high school students play video games for more than 1 h on weekdays [46]. Thus, video game–based training will become more popular in the future.

On the other hand, a series of meta-analyses [47] found only small or null effect sizes in three models examining correlations between video game skills and cognitive ability, differences in cognitive ability between game players and non-players, and the effects of video game–based training on cognitive ability, respectively. Thus, examination of the clinical effects of real or virtual training using board games may provide more appropriate information for discussion of the advantages and disadvantages of each style of board game for future applications in clinical settings. A recent assessment of cognitive science research on board game playing [48] highlighted six suggestions for future studies: 1) do not forget about chess (i.e., a traditional board game for which large amounts of data have been collected), 2) look beyond action games and chess, 3) use optimal play to understand human play and players, 4) investigate social phenomena, 5) raise the standards for studies investigating game play as treatment, and 6) talk to real experts.

## Conclusions

Although the number of studies investigating board game use remains limited, interesting findings have recently been obtained in terms of brain function, cognitive effects, and health-related lifestyle modification. Board games may also be applicable as educational tools for health professionals. Although a systematic review [7] neither confirmed nor refuted the utility of game playing as a teaching strategy for health professionals, these findings were published in 2013 and additional high-quality studies have been reported since then. Thus, it is time to re-evaluate the usefulness of games and gamifications following technological advances made in modern society. Clinical medicine is closely linked to a public health approach, and medical practices should be undertaken within the limited human, time, and financial resources available [49]. In this sense, appropriate health education programs with a board game component would be useful for both preventive and therapeutic intervention for cognitive-behavioral functioning (e.g., ADHD and dementia), psychological conditions (e.g., depression and anxiety disorders), and life-style diseases (e.g., metabolic syndromes and smoking-related diseases).

## Abbreviations

(f)MRI: (functional) Magnetic resonance imaging
AD: Anno Domini
ADHD: Attention-deficit/hyperactivity disorder
AI: Artificial intelligence
BDNF: Brain-derived neurotrophic factor
EEG: Electroencephalographic
HIV: Human immunodeficiency virus
ICT: Information and communication technology
RCT: Randomized controlled trial
